# Comparative Evaluation of Anticorrosive Properties of Mahaleb Seed Extract on Carbon Steel in Two Acidic Solutions

**DOI:** 10.3390/ma12183013

**Published:** 2019-09-17

**Authors:** Aisha A. Ganash

**Affiliations:** Chemistry Department, Faculty of Science, King Abdulaziz University, Jeddah 23714, Saudi Arabia; aganash@kau.edu.sa

**Keywords:** green inhibitor, mahaleb, carbon steel, Langmuir isotherm, physisorption

## Abstract

Aqueous extract solution of Mahaleb seed (ASMS) was prepared using a simple and safe method. ASMS was tested to examine its potential to act as a green corrosion inhibitor for preventing the dissolution of Carbon steel in highly concentrated corrosive 2 M H_2_SO_4_ and 2 M H_3_PO_4_ using an electrochemical polarization Tafel plot and electrochemical impedance spectroscopy. ASMS provided a slight increase in the inhibition efficiency of H_3_PO_4_ (89%) compared with H_2_SO_4_ (86%). Fourier transform infrared spectroscopy (FTIR) and electronic scanning microscopy (SEM) were used to prove that adsorption of ASMS occurred on the metal surface. The thermodynamic adsorption and thermodynamic activation parameters were calculated at a range of concentrations and temperatures. The physisorption of ASMS followed the Langmuir adsorption isotherm (R^2^ = 0.98). Finally, the role of ASMS as a protection mechanism was discussed.

## 1. Introduction

A significant portion of the annual costs of the oil-production concerns worldwide is assigned to solving corrosion problems. Thus, many kinds of research have been done to solve difficulties in corrosion.

Laboratory studies continue to find viable solutions to control the corrosion process as research and methods are expanded to reduce its severity. These studies have included various methods such as anodic protection, cathodic protection, the use of inhibitors, and conductive coating.

Inhibitors are chemical compounds that are added in small proportions to a solution so that it is adsorbed on the surface of the metal and forms a thin film that prevents direct contact between the metal and corrosive solution [1].

However, Fekry et al. [2] pointed out that the addition of organic compounds with rich electron heteroatoms such as P, O, N, and S is an excellent way to inhibit the corrosion of steel materials in acid solution [3,4,5,6], although the efficiency of the inhibitory process depends mostly on the types and structures of adsorbed film on the metal surface [7,8].

Chemical compounds are a highly effective way to discourage corrosion, but may have a toxic effect, which can affect human lives. Therefore, a recent trend is the use of eco-friendly materials, such as plant extracts containing a mixture of compounds, which can be adsorbed on the surface of the metal and work very efficiently to control the process of corrosion, and at the same time, do not harm the environment. The plant extracts have been used in different areas, such as for the green synthesis of metal nanoparticles [9], antimicrobial prevention, and corrosion inhibition [10,11,12,13,14]. Studies in this field have used extracts of different parts of plants, including the seeds, stalks, or flowers of the plant, in various media, for example, acidic, basic, or neutral solution. A previous study reported that Ruta extract solution provides functional inhibition for steel that reaches 87% in 1 M HCl [15]. Bammu et al. investigated the effect of Chenopodium Ambrosioides extract solution to reduce the corrosion rate of C-steel in 0.5 M H_2_SO_4_ [16]. Another survey, conducted by Anupama showed that Phyllanthus amarus extract is an excellent mixed-type inhibitor for mild steel in 1 M HCl [17].

The seeds of Mahaleb are very prevalent in the Middle East. These oval, pointed seeds are golden brown with a soft texture and are used in several areas, such as for flavoring during cooking or as hair fresheners. They are also an important method of tooth pain reflief in infants during teeth protrusion and to reduce the resulting fever during teething.

Steel and its various alloys are essential metals that are used in construction and different industries because of their hardness and strength. Polished steel can resist corrosion in middle-acidic circles, but unfortunately, if the acidicity increases, this metal fails to sustain resistance.

The primary objective of this study is to examine and compare the ability of Mahaleb seed extract (ASMS) to control the corrosion of carbon steel in corrosive 2 M H_2_SO_4_ and 2 M H_3_PO_4_. Electrochemical techniques such as potentiodynamic anodic and cathodic polarization (PDP) and electrochemical impedance spectroscopy (EIS) were carried out in a temperature range of 298–328 °K in the presence of different concentrations of ASMS.

## 2. Experimental Methods

### 2.1. Materials and Instrumentation

Chemical reagents, such as H_2_SO_4_ and H_3_PO_4_, were used as received without further distillation. ASMS seeds were bought from the local market in Jeddah, and the carbon steel was cut as a rod with a 1 cm diameter and 5 cm length. The working electrode area was adjusted using a Teflon cap, which surrounded the rod and restricted the required area to 1.09 cm^2^ on the top of the electrode. The working electrode was connected to stainless steel wire to promote the transfer of current through the electrochemical cell.

The electrochemical measurements were performed in aerated solution using an ACM GillAC single-channel potentiostat/galvanostat (ACM Instrumrnts, Sharjah. United Arab Emirates) connected to a polarization cell with three nicks for Ag/AgCl (3 M KCl, Metrohom, Herichaut, Switzerland) as the reference electrode, a Pt rod (L = 5 cm and OD = 0.2 cm, Metrohom) as the auxiliary electrode, and carbon steel as the working electrode.

Fourier transform infrared spectroscopy (FTIR) was studied in the range 500–4000 cm^−1^ using the Perkin Elemer Inc Instrument (Akron, OH, USA), while electronic scanning microscopy (SEM) was performed using FESEM (7600F Jeol JSM, Tokyo, Japan).

The extract solution was prepared by boiling 20 g of the golden-brown seeds with 300 mL of distilled water for two hours, utilizing the steam evolved from the water bath at 100 °C without direct immersion in the water to reserve all components from evaporation. The hot solution was cooled overnight and filtered using a Buchner Funnel. The extract was saved in the fridge to keep it from rotting.

### 2.2. The Electrochemical Measurements

The corrosion test was performed by forming anodic and cathodic Tafel plots in the presence and absence of different concentrations of ASMS in acid solution in a temperature range of 298–328 °K. Measurements were conducted after one hour to establish the equilibrium potential (*E*_eq_) of the electrode in the corrosive acid. The working electrode was polished with different grades of sandpaper up to 1200, was washed carefully with excess tap water, distilled water, and finally, with ethanol. The clean electrode was allowed to dry at room temperature before each measurement. The electrode was polarized in the potential range of ±200 mV versus *E*_eq_ with a scan rate of 1 mV/s.

Electrochemical impedance spectroscopy (EIS) was carried out potentiostatically in the frequency range of 30 kHz to 0.1 Hz versus *E*_eq_ with an amplitude of 10 mV/s.

## 3. Results and Discussion

### 3.1. Effect of Inhibitor Concentration

#### 3.1.1. Electrochemical Impedance Spectroscopy

**Figure 1:**
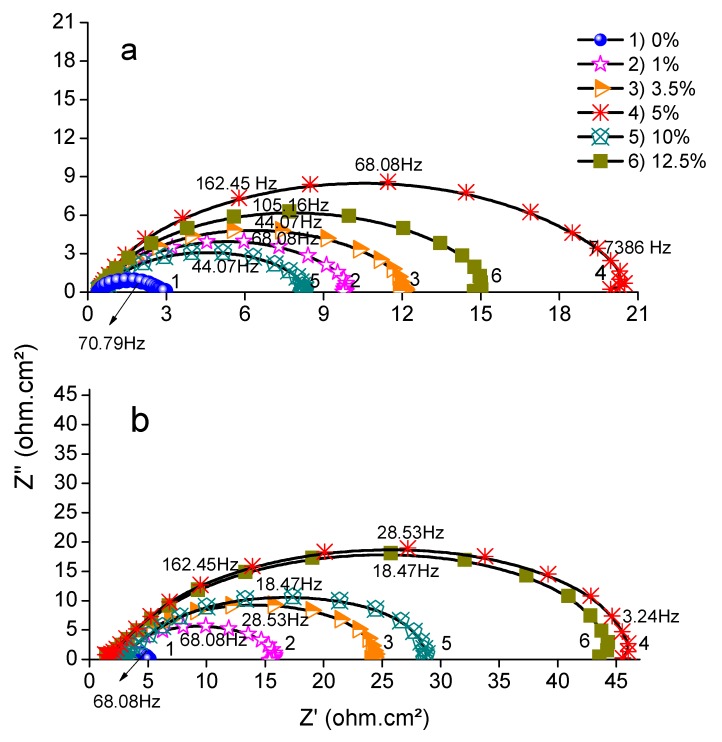
Nyquist plots for Carbon steel in (**a**) 2 M H_2_SO_4_ and (**b**) 2 M H_3_PO_4_ in the absence and presence of different concentrations of Mahaleb seed extract (ASMS) at 298 °K.

The study was carried out at a constant temperature of 298 °K, and the effects of different amounts of ASMS in 2 M H_2_SO_4_ and 2 M H_3_PO_4_ solution were determined. EIS Nyquist plots are depicted in Figure 1a,b. The Nyquist diagram shows a depressed semicircle with a capacitive loop in the high-frequency region. Deviation from the ideal semicircle occurred due to the roughness and heterogeneity of the spreading of the active site [18,19]. The figure shows that the shape of the curve does not differ with or without the presence of ASMS; this is strong evidence that the addition of different concentrations of ASMS did not affect the corrosion reaction mechanisms of the carbon steel. It is clear that the semicircle diameter increases as the concentration level increases from 1% to 5%. However, a higher concentration (10%) causes a lower inhibition efficiency. The decrease in the inhibition efficiency in higher concentrations may be due to the high concentration of ASMS causing dispersion and randomization of inhibitor molecules that are adsorbed on the electrode surface as a result of contention and rivalry between ASMS and the corrosion product. In this regard, a concentration of 5% is considered the optimum concentration in this study, and the maximum inhibition efficiency is 88% in the case of H_2_SO_4_ and 92% for H_3_PO_4_. The inhibition efficiency (σ) was calculated from Equation (1).

(1) $$\sigma (\%)=(\frac{R_{p}-R_{p}^{o}}{R_{p}})\;\times \;100$$

where R°_p_ is the polarization resistance of the blank, and R_p_ is the polarization of the solution. 

One of the most crucial regions of EIS is the direct construction that exists between the nature of a real system and that of an idealized model circuit consisting of discrete electrical components. Therefore, EIS data were transferred to a simulation software program (Zsim) to obtain the best-fitting equivalent circuit (EC) and to permit the calculation of kinetic parameters that are compatible with the physical and chemical properties of the system. The kinetic parameters were obtained by fitting with an appropriate equivalent circuit {R_s_(CPE_f_ (R_f_ (CPE_dl_R_ct_)))}, and the evaluated parameters are summarized in Table 1. The electrical circuit consists of solution resistance (R_s_), film resistance (R_f_), and charge transfer resistance (R_ct_). The summation of R_f_ and R_ct_ gives the polarization resistance (R_p_) [20]. The change in the Rs value may be attributed to the solution trapped inside the pores of the film, which produces higher resistance [21]. However, future investigation will be carried out to understand the variation in Rs. On the other hand, (CPE)_f_ and (CPP)_dl_ represent the constant phase element of the film and the electrical double layer, respectively. The CPE consists of two parts, the exponent (n) and the pseudocapacitance (Q), which consider the replacement of the pure capacitance (C) and represent the deviation from ideal dielectric behavior due to surface heterogony [22]. As discussed elsewhere, the electrical double layer capacitance (C_dl_) is a measure of the adherence of the inhibitor molecules on the electrode surface. A lower value of C_dl_ represents the best adherence and vice versa. The increase in R_p_ with concentration is attributed to the increasing thickness of the film adsorbed on the metal surface, which reduces the charge transfer between the steel surface and corrosive acidic medium. C_dl_ is obtained from the frequency corresponding to the maximum value of the imaginary part of the Nyquist plot (w_max_). It decreases as the concentration of the inhibitor increases; this can be explained by the replacement of water molecules with inhibitor molecules, which are adsorbed on the metal surface and form a compact layer, decreasing the free area of metal in contact with the solution. As a result, the dialectical constant reduces due to the increase in the double layer thickness, increasing the protection from the penetration of corrosive ions [23]. C_dl_ was calculated using Q and w_max_ by applying Equation (2):

Cdl = Q (2π w_max_)*^n^*^−1^ (2)

**Figure 2:**
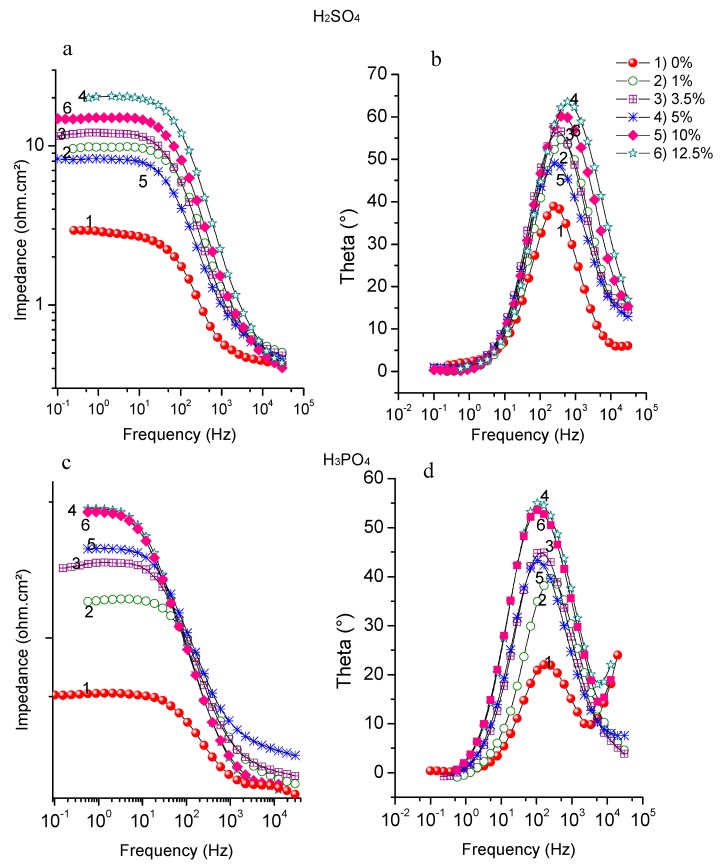
Bode plots (**a**) and (**b**) in H_2_SO_4_, (**c**) and (**d**) in H_3_PO_4_ for Carbon steel in the presence in absence and presence of different concentrations of ASMS at 298 °K.

The Bode impedance curve was also studied and is shown in Figure 2a,b. The low-frequency region in the impedance/frequency curve corresponds to the polarization resistance and electrolyte resistance, while the high-frequency part corresponds to the individual electrolyte resistance. So, an increase in area under low frequency occurs when a higher amount of ASMS is adsorbed on the metal surface, consequently increasing the amount of protection. The phase angle/frequency plot supports the previous result; hence, the increase in the area of low frequency indicates a rise in R_p_ with an increased ASMS concentration. However, an increase in the area of high frequency indicates a highly absorbed molecule on the steel surface [24].

It is noteworthy that all curves in the phase angle plots shifted as the inhibitor concentration increased; this indicates that a staple and compact layer of ASMS film was adsorbed on the metal surface [25]. On another hand, all curves were less than 90°; this is ascribed to a deviation from the ideal capacitor behavior, as described elsewhere [26].

#### 3.1.2. Tafel Polarization

**Figure 3:**
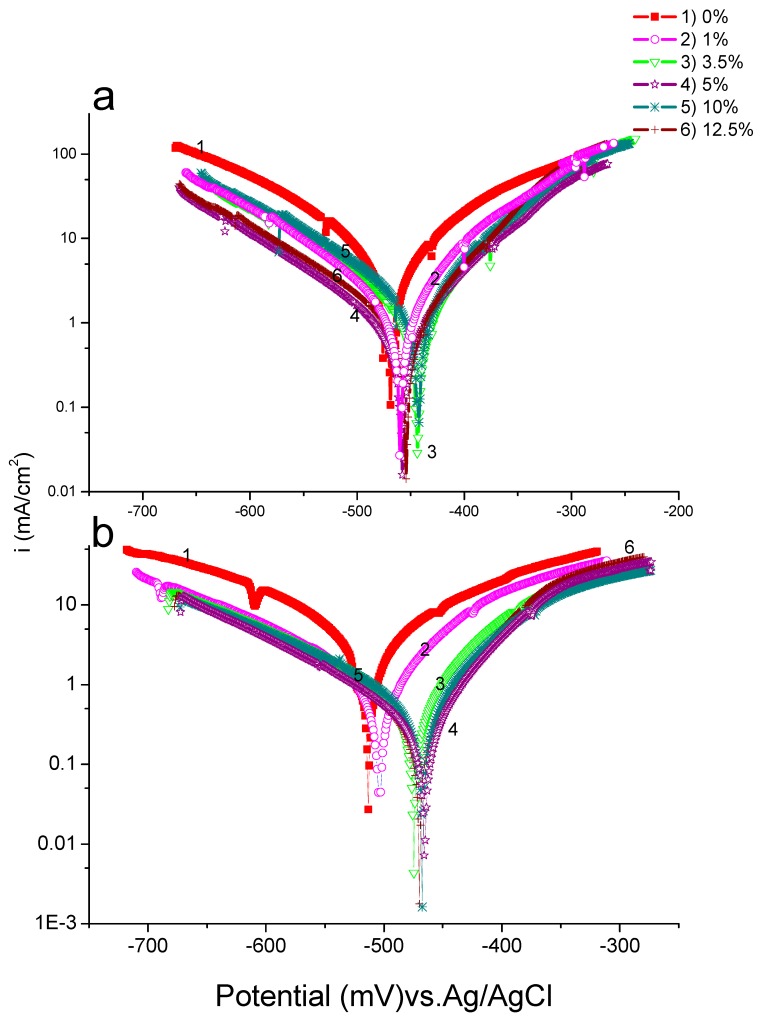
Potentiodynamic polarization curves for Carbon steel in (**a**) 2 M H_2_SO_4_ and (**b**) 2 M H_3_PO_4_ in the absence and presence of different concentrations of ASMS at 298 °K.

The potentiodynamic polarization (PDP) was measured for different amounts of ASMS, as depicted in Figure 3 for 2 M H_2_SO_4_ and 2 M H_3_PO_4_. The Tafel plot in the anodic direction shows active dissolution of the metal in this region, and no passive behavior occurred in the potential range under study. The Tafel corrosion current density (i_corr_) reduced by 7.107 and 4.112 mA/cm^2^ in H_2_SO_4_ and H_3_PO_4_, respectively, as the ASMS concentration increased, reaching minimum values in the presence of 5% of the inhibitor (0.982 mA/cm^2^ in H_2_SO_4_ and 0.426 mA/cm^2^ in H_3_PO_4_), which suggests that ASMS was adsorbed at the steel/acid solution interface and blocked the active site, providing more protection. In addition, the corrosion potential (*E*_corr_) shifted to a more noble value (positive value) compared with that of the bare metal. The parallel shape for the anodic and cathodic parts suggests that the addition of ASMS reduced the hydrogen evolution and oxidation of the metal. Additionally, the existence of the inhibitor molecules did not affect the corrosion reaction mechanism [27,28]. Since both anodic and cathodic reactions were affected by the addition of ASMS, this type of inhibitor can be categorized as a mixed-type inhibitor with an anodic predomination effect. Furthermore, the symmetry of the energy barrier in the anodic and cathodic directions can be expressed by evaluating the cathodic and anodic Tafel slopes, βc and βa. In this context, the shift in *E*_corr_ for the entire range of concentrations is less than ±85 mV; this value is considered to be the benchmark for classification of this type of inhibitor [29]. The electrochemical corrosion parameter was evaluated using the Tafel slope analysis method, and the results are listed in Table 2. The percent of corrosion inhibition efficiency (σ) was calculated using Equation (3).

(3) $$\sigma (\%)=(\frac{i_{\mathrm{corr}}^{o}-i_{\mathrm{corr}}}{i_{\mathrm{corr}}^{o}})\;\times \;100$$

where i_o_ and i are the corrosion current density for blank and inhibited solutions, respectively. 

As reported previously [30], the corrosion parameter can be estimated to be around ±15 mV of *E*_corr_ or in the Tafel range of ±250 mV of *E*_corr_. It is noteworthy that in the potential range between *E*_corr_ and −300 mV versus Ag/AgCl, ASMS provided good inhibition that reaches 86% and 89% in H_2_SO_4_ and H_3_PO_4_, respectively, owing to the formation of an adsorbed film on the inhibitor. Meanwhile, at potentials above −300 mV, the inhibition efficiency was reduced. This may have been due to the increase in the dissolution of steel electrode, leading to the desorption of ASMS molecules from the surface of the metal. At this stage, the desorption process dominated instead of the adsorption process [31,32]. The Tafel plot exhibited two regions; at the low overpotential region, anodic and cathodic reactions were under activation control, whereas at the high overpotential region, the transfer of ions towards metal surface was the rate determining step, and a diffusion current was produced [30]. 

**Figure 4:**
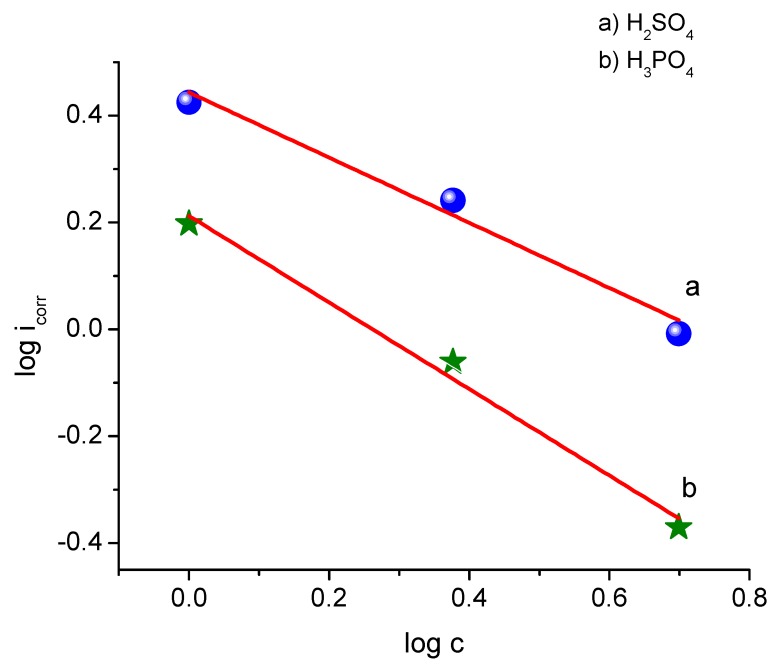
The variation of log *i*_corr_ with log ASMS concentration at 298 °K.

The kinetic relationship between log i_corr_ against log C_inh_, represented in Figure 4, depends on Equation (4).

(4) $$\log i_{\mathrm{corr}}=\log K+\beta \log C_{\mathrm{inh}}$$

where β is a constant measure of the effectiveness of adsorbed ASMS molecules.

The negative value of β for both acids indicates that the corrosion rate decreases with a gradual increase in the ASMS concentration. It is important to note that β is equal to −0.810 in H_3_PO_4_, which is more negative than the value found in H_2_SO_4_ (−0.609). It has been reported that a higher negative value of β reflects better inhibition properties. 

The constant K represents the value of the corrosion rate when the concentration is equal to the unity. In comparison, the value of k from plotting was shown to be equal to 2.77 and 1.63 in H_2_SO_4_ and H_3_PO_4_, respectively; these values are very similar to the values predicted from the Tafel slope analysis, 2.66 and 1.59 mA/ cm^2^, for both acids. In another study, it was suggested that the value of K describes the corrosive effect of the corrosive environment on the metal [33]. Based on this study, H_2_SO_4_ acid is considered to be more corrosive than H_3_PO_4_ acid, even in the presence of the same quantity of ASMS.

### 3.2. Potential-Time Measurements

**Figure 5:**
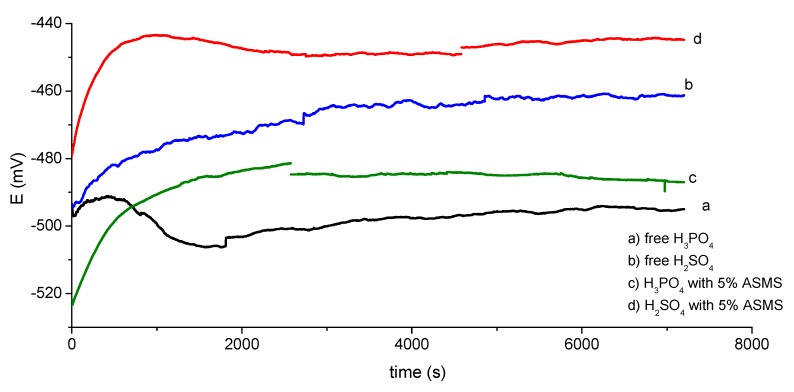
The potential-time curve for different solutions for two hours at 298 °K.

Figure 5 shows the variation in the open circuit potential (E_ocp_) with time for 2 h for 2 M H_2_SO_4_ and 2 M H_3_PO_4_ acid with and without 5% ASMS. In free H_2_SO_4_ acid, there was a regular increase in E_ocp_ in the positive direction, indicating the passivation of the steel surface due to the formation of the oxide layer. At 49 min, the potential was almost constant, and a steady state was reached at around −461 mV. While in cases of free H_3_PO_4_, there was a small increase in E_ocp_ due to the formation of the passive layer; this layer was stable for a concise period of time (≈8 min), followed by a regular decrease in E_ocp_, which continued steadily during the measurement and reached −495 mV. The effect of the addition of 5% ASMS in both acids was similar, since the rapid increase of E_ocp_ with the addition of the inhibitor molecule indicated a fast adsorption process and formation of a thin film of ASMS on the steel surface. The E_ocp_ was shifted to a more positive value, which provides strong evidence that the anodic process is more affected by ASMS adsorption.

### 3.3. The Thermodynamic Adsorption Parameter

**Figure 6:**
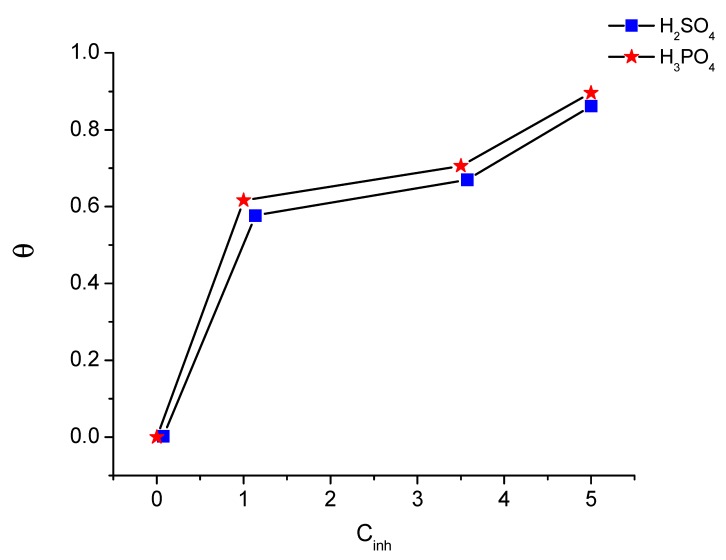
S-shape of θ at different ASMS concentrations.

The adsorption process of the inhibitor on the metal surface is an essential factor in clarifying the mechanism of inhibition. The first step in the adsorption process is the approach of the ASMS molecule to the surface of the metal and the rejection of the adsorbed water molecule; this process is due to the interaction forces (energy) between ASMS and the steel surface being higher than the interaction forces between the water molecules and the steel surface [34]. The adsorption isotherm is considered the best method to express the adsorption process quantitively; this isotherm is representative of the coverage degree (θ) versus different concentrations of ASMS at a constant temperature. The produced curve (Figure 6) flows in an S-curve shape, since the curve trends gradually upwards and then stays almost constant, which implies the adsorption of a monolayer of ASMS molecules on the metal surface. A value of θ was calculated depending on the PDP measurement using Equation (5).

(5) $$\theta =\frac{\sigma }{100}$$

**Figure 7:**
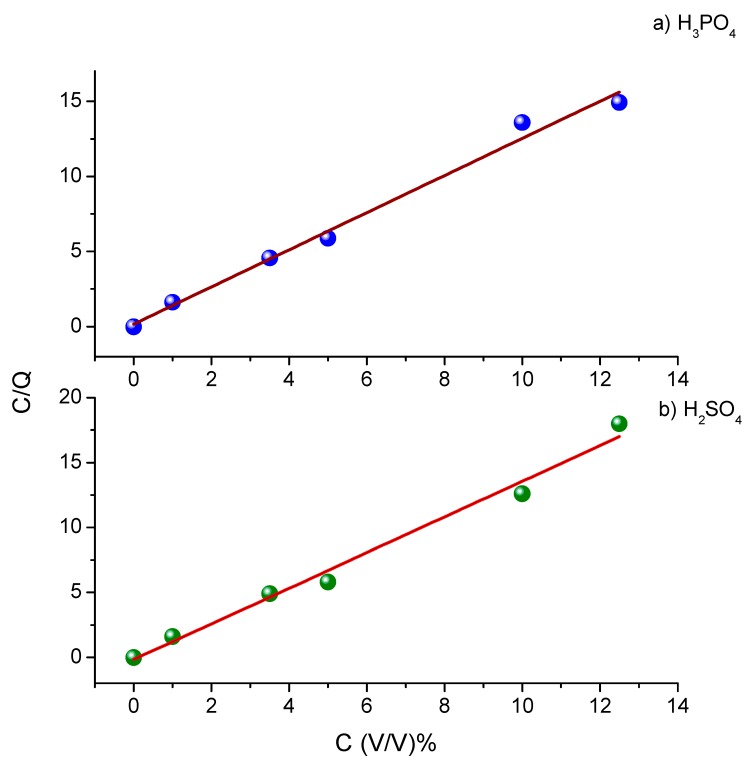
Langmuir adsorption isotherm for (**a**) H_3_PO_4_ and (**b**) H_2_SO_4_ with different concentration of ASMS.

The value of the regression coefficient (R^2^ = 0.98) of the Langmuir adsorption isotherm [35,36] (Equation (6)) is considered the best-fit isotherm, as shown in Figure 7a,b.

(6) $$\frac{C_{\mathrm{inh}}}{\theta }=\frac{1}{k_{\mathrm{ads}}}+C_{\mathrm{inh}}$$

where k_ads_ is the Langmuir adsorption equilibrium constant, which represents the equilibrium constant between the adsorption and desorption processes.

Since the adsorption follows the Langmuir isotherm, this emphasizes that ASMS is adsorbed physically as a monolayer on steel surface [13,37]. Moreover, the slope of the isotherm is equal to 1.3 with H_2_SO_4_ and 1.24 with H_3_PO_4._ These values are close to unity, which means each active site on the steel surface is occupied by only one ASMS molecule (i.e., the monolayer of ASMS adsorbed on the steel surface). Depending on the extract, various organic compounds may be present, which are adsorbed differently on the metal surface. These compounds mostly interact with each other via attraction or repulsion. Moreover, these compounds may adsorb on both anodic and cathodic active sites on the surface. All the previous WHAT lead to deviation of the slope from unity (ideal Langmuir slope).

The spontaneity of adsorption is corroborated by the related K_ads_ with Gibbs free energy ΔG_ads_ using Equation (7):

(7) $$\Delta G_{\mathrm{ads}}=RT\left[\ln\frac{{\left(55.5\right)}^{-1}}{k_{\mathrm{ads}}}\right]$$

where the value 55.5 is a concentration of the H_2_O molecule

The results of the thermodynamic adsorption parameter are tabulated in Table 3. The negative value of ΔG_ads_ caused the spontaneous nature of the adsorbed molecules on the steel surface. Since ΔG_ads_ was less than 20 kJ/mol, this suggests that the physical adsorption occurred through the electrostatic attraction between ASMS molecules and the metal [38]. As described previously, physisorption is the electrostatic attraction between charged metal and an inhibitor molecule, while chemisorption describes the sharing of electron pairs between an inhibitor and vacant d-orbitals on the metal.

The heat of adsorption ΔΗ_ads_ was calculated for 5% ASMS at different temperatures using Equation (8):

(8) $$\frac{\theta }{1-\theta }=AC_{\mathrm{inh}}exp\left(\frac{-\Delta H_{\mathrm{ads}}}{RT}\right)$$

The plot of ln ($\frac{\theta }{1-\theta }$) versus (1/T), as shown in Figure S1, produced an ΔΗ_ads_ value equal to −100 kJ/mol with H_2_SO_4_ and −52.7 kJ/mol with H_3_PO_4_. The negative values confirm the physical adsorption of ASMS on the steel surface [39]. Referring to the previous investigation, the chemisorption is related to an endothermic reaction, while the exothermic reaction is related to the physisorption process [40]. As expected, the entropy of adsorption (ΔS_ads_) was negatively charged, due to the exothermic process, which was accompanied with reduced entropy. ΔS_ads_ can be estimated using Equation (9), which relates the ΔG_ads_ and ΔΗ_ads_:

(9) $$\Delta G_{\mathrm{ads}}=\Delta H_{\mathrm{ads}}-T\Delta S_{\mathrm{ads}}$$

It is noteworthy that the calculated values of K_ads_ and ΔG_ads_ were similar for the two acids, suggesting that the adsorption of ASMS molecules converges in both acids. All estimated parameters are reported in Table 3.

As documented previously, it is difficult to elucidate the adsorption behavior for the natural product inhibitor using the calculated ΔG_ads_. This difficulty is because the chemical nature as well as the molar mass of the adsorbent component have not been accurately determined. For this reason, more parameters should be calculated, such as the energy of activation (E_a_).

### 3.4. Effect of Temperature Change

**Figure 8:**
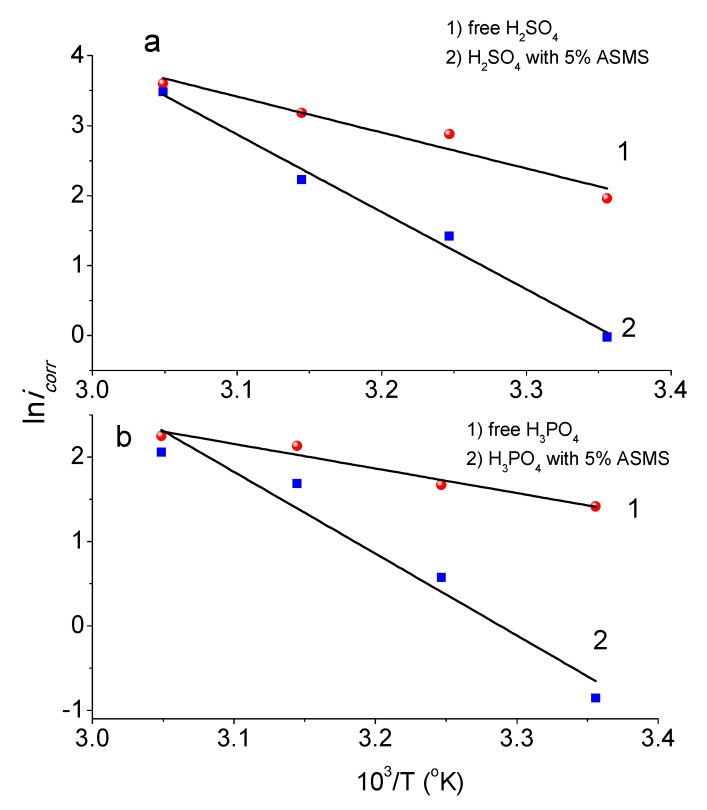
Arrhenius relationship for H_3_PO_4_ (**a**) and H_2_SO_4_ (**b**) with and without 5% ASMS.

The effect of temperature on the corrosion process is one of the most important factors which plays a key role in understanding the kinetics of the reaction. The temperature change is considered to have a profound affect because it leads to many changes at the metal surface (e.g., rabid etching, as well degradation or rearrangement of the inhibitor and desorption of inhibitor molecule from the metal surface) [41,42]. Therefore, this section deals with the study of the corrosion process in the temperature range 298–328 °K in the presence and absence of 5% ASMS in 2 M H_2_SO_4_ and 2 M H_3_PO_4_. As described previously, the Arrhenius relationship (Figure 8a,b) relates E_a_ with the natural logarithm of the corrosion rate (express in i_corr_ term) using Equation (10):

(10) $$i_{\mathrm{corr}}=Aexp\left(\frac{-E_{a}}{RT}\right)$$

where A is a pre-exponential factor, and E_a_ was calculated from the slope of plotting ln i_corr_ against 1/T.

The other activation parameters, such as the heat of activation ΔH*, the entropy of activation ΔS*, and the free energy of activation ΔG* were estimated using transition state theory (Equation (11)) and the fundamental equation (Equation (12)) through thermodynamic chemistry:

(11) $$\ln\left(\frac{i_{\mathrm{corr}}}{T}\right)=(\left[\ln\left(\gamma^{*}\right)+\left(\frac{\Delta S^{*}}{R}\right)]-\frac{\Delta H^{*}}{RT}\right)$$

(12) $$\Delta G^{*}=\Delta H^{*}-T\Delta S^{*}$$

where γ* is the collision frequency, which is equal to $\frac{RT}{Nh}$, ΔH* and ΔS* were calculated from the slope and the intercept produced from plotting ln (i_corr_/T) versus 1/T, as shown in Figure S2, and the parameters are listed in Table 3.

In all case of acids, the E_a_ for the inhibited solution with 5% ASMS (≈79.7 kJ/mol in M H_2_SO_4_ and ≈80.6 kJ/mol in H_3_PO_4_) was greater than the E_a_ in uninhibited solution (≈42.7 kJ/mol in M H_2_SO_4_ and ≈24.2 kJ/mol in H_3_PO_4_), and there was a decreasing in σ with an increasing temperature. Li et al. [43] suggested that a higher energy barrier is needed under strong physical adsorption for the corrosion reaction to reach the product. So, the adsorption of ASMS on steel surface is classified as a physisorption process, produced from the simple electrostatic attraction between the metal surface and ASMS molecule.

However, the ΔH* for the free acid and inhibited system was a positive value, associated with the endothermic reaction. Guan and coworkers [44] demonstrated that the dissolution of metal is an endothermic reaction. Thus, the high positive value of ΔH* represents a more endothermic reaction (i.e., more energy needs to be absorbed). The presence of 5% of inhibitor molecules caused a rise in ΔH* compared with the free acid.

It is interesting to note that for both types of acid used in this study, the E_a_ was higher than the ΔH*, leading to the evolution of hydrogen gas and decreasing the solution volume during the corrosion process. The difference value (E_a_- ΔH*) was around 2.59 kJ/mol, which was similar to the RT value (≈2.69 kJ/mol). This provides substantial evidence that the corrosion reaction is unimolecular, based on Equation (13) [45]:

(13) $$E_{a}-\Delta H^{*}=RT$$

The positive value of ΔS* in the presence of 5% ASMS was unexpected, since the adsorption of the inhibitor is an exothermic process (ΔΗ_ads_ is negative) that requires the replacement of solvent molecules and is expected to increase the order of the solute (decrease ΔS*) and decrease the order of the solvent (increase ΔS*). A possible explanation for this might be that ΔS* represents the summation of the entropy of the solvent and solute molecules (S*_solvent_ + S*_solute_). Thus, the increase in ΔS* is due to the rise in S*_solvent_, thereby increasing the entropy when transfering from the reactant, to form an activated compound at the steel/ASMS interface, which represents the driving force of the adsorption process. Although these results differ from some published research, they are consistent with the research presented in [46,47,48].

Finally, the higher value of ΔG* in the presence of 5% of ASMS compared with free acid provides strong evidence of the decreased stability of the corrosion-activated compound with an increasing temperature.

### 3.5. FTIR Spectra Analysis

**Figure 9:**
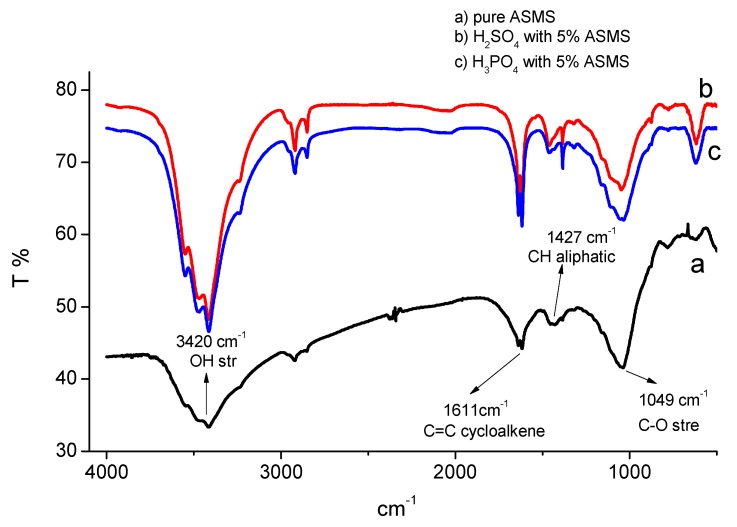
Fourier transform infrared spectroscopy (FTIR) spectra of different solutions (**a**) pure ASMS, (**b**) H_2_SO_4_ with 5% ASMS and (**c**) H_3_PO_4_ with 5% ASMS.

The FTIR spectra measurements were performed with a KBr belt for pure ASMS and for carbon steel immersed in both 2 M H_2_SO_4_ and 2 M H_3_PO_4_ with 5% ASMS for 24 h at 298 °K, to demonstrate the adsorption of ASMS molecules on the metal surface, as depicted in Figure 9.

The FTIR spectra provided all of the characteristic bands for the different compounds in ASMS (Figure 9a). The band at 3420 cm^−1^ refers to the OH stretching vibration, which may interfere with CH aromatics. However, the broadening of the band may be attributed to the formation of a hydrogen bond between OH and carbonyl group of the inhibitor [11]. The band at 1611 cm^−1^ is attributed to the C=C cycloalkene bond. Moreover, CH appeared at around 778 cm^−1^. The C–O stretching band was detected at around 1049 cm^−1^, while the band at around 1427 cm^−1^ is attributed to the CH aliphatic. The spectra of the adsorbed ASMS in 2 M H_2_SO_4_ (Figure 9b) and 2 M H_3_PO_4_ (Figure 9c) solution show the same distinguishing bands, with small shifts due to the adsorption of the inhibitor molecule on the steel surface. The sharpness of the OH peak after inhibitor adsorption can be explained by the disappearance of the hydrogen bond between the inhibitor molecule, which confirms the bond formation between the inhibitor and the metal surface during the adsorption process [11].

### 3.6. SEM Image Investigation

**Figure 10:**
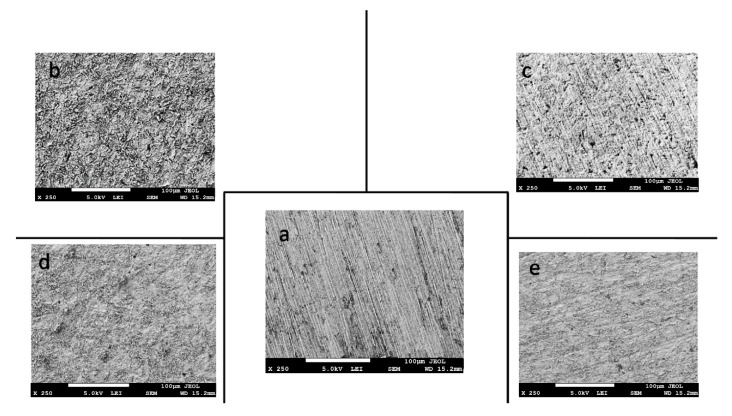
SEM images for (**a**) polished steel, (**b**) steel immersed in free 2 M H_2_SO_4_, (**c**) steel immersed in free 2 M 2 M H_3_PO_4_, (**d**) steel immersed in 2 M H_2_SO_4_ in the presence of 5% ASMS, and (**e**) steel immersed in 2 M H_3_PO_4_ in the presence of 5% ASMS.

The SEM images are shown in Figure 10a–e for clean polished steel, steel immersed in blank acids, and steel immersed in acids with 5% ASMS. Figure 10a represents the metal surface before immersion in the corrosive acid. It is clear from the figure that the surface is smooth except for some scratches, due to the polishing process. However, Figure 10b,c show the metal after immersion in 2 M H_2_SO_4_ and 2 M H_3_PO_4_ for one hour, respectively. The metal surface is rough due to the dissolution of the metal, and the pits are spread on the metal surface. It is noteworthy that H_2_SO_4_ highly affected the surface compared with H_3_PO_4_. Figure 10c,d show images of the steel surface in the presence of ASMS. The roughness of the surface was reduced, and the surface became smoother due to the adsorption of a thin film of the ASMS molecules on the metal surface, which provided a protective layer and diminished the corrosion process.

### 3.7. The Role of ASMS in Corrosion Inhibition

To explain the inhibition mechanism, it is necessary to know the chemical composition of ASMS. In 2016, Mead and coworkers analyzed ASMS using gas–liquid chromatography and demonstrated that ASMS contains Stigmasterol (4.64), Cholesterol (10.31%), β-sitosterol (10.57%), Heneicosane (62.57%), and fatty acids such as Timnodonic (33.07%), linoleic (24.35%), and oleic (28.71%) acids, as shown in Table 4.

It was suggested [49] that all constituents of the inhibitor support each other in the inhibition process, so it does not make sense to determine which specific compound is adsorbed on the metal surface first. Thus, it is more convent to consider ASMS as a collection of active components that work together to reduce the corrosion process. It has been identified that major factors that may affect the adsorption of organic compounds on metal surfaces include the type of metal, the type of corrosive medium, the chemical composition of the inhibitor, and the interaction between the adsorbent and the inhibitor [50].

Mcfferty [51] explained that the inhibitor molecules reduce the corrosion rate of the metal in corrosive solution by affecting the kinetic process of metal oxidation or by changing the electrochemical behavior. The inhibitor molecules are adsorbed immediately when the electrode is immersed in the solution, and thin film isolate forms on the metal due to the direct contact with the corrosive ions. Physical adsorption is considered to be the first step in the adsorption of the organic compound due to the short time period required for this adsorption process to reach equilibrium.

It has been described that steel carries a positive charge in acidic medium, which attracts the negative anions of the electrolyte, either SO_4_^−2^ or PO_4_^−3^. The adsorbed layer becomes negatively charged and quickly attracts the cations of the inhibitor. So, the anion is considered to be a connector between the two positive dipoles (metal and ASMS molecules) that forms a thin film of ASMS molecules and prevents greater passage of the corrosive electrolyte to the electrode surface.

In general, the remarkable increase of (σ) in H_3_PO_4_ may be attributed to the higher negative charge for PO_4_^−3^ than for SO_4_^−2^, which allows greater adsorption on the positively charged electrode and generates extra negative charge in the solution; this leads to more synergistic adsorption of ASMS molecules on the steel surface.

## 4. Conclusions

ASMS was prepared and examined to determine its potential to act as a green corrosion inhibitor in 2 M H_2_SO_4_ and 2 M H_3_PO_4_ for Carbon steel at different temperatures. In this investigation, electrochemical methods such as PDP and EIS were used to determine the corrosion rate and, consequently, the inhibition efficiency in both acids. The resulting polarization curves suggest that ASMS acts as a mixed-type inhibitor. It was shown that ASMS exhibits better protection in H_3_PO_4_ compared with H_2_SO_4_ acid solution at a constant temperature. The adsorption process obeys the Langmuir isotherm, and it is a physisorption process since the inhibition protection decreases as the temperature increases.

## Figures and Tables

**Table 1:** Electrochemical impedance parameters in the presence of various concentrations of ASMS.

Solution	C_ASMS (*v*/*v* %)_	R_s_ (Ω cm^2^)	R_f_ (Ω cm^2^)	C_dl_ (μF cm^−2^)	R_ct_ (Ω cm^2^)	R_p_ (Ω cm^2^)	σ
**2 M H_2_SO_4_**	0.00	0.38	2.28	2767	0.08	2.37	0.00
	1.00	0.50	6.77	2406	2.74	9.51	75.2
	3.50	0.49	9.97	1897	1.86	11.8	80.0
	5.00	0.41	11.1	1194	9.26	20.4	88.4
	10.0	0.58	7.91	6682	0.03	7.94	70.2
	12.5	0.40	0.19	2010	14.8	15.0	84.3
**2 M H_3_PO_4_**	0.00	1.59	3.61	85.9	0.05	3.67	0.00
	1.00	1.79	1.05	20.3	14.2	15.2	75.9
	3.50	1.78	2.01	20.0	14.3	16.3	77.5
	5.00	1.70	44.9	3.20	0.37	45.3	91.9
	10.0	2.02	1.09	168.0	27.1	28.2	86.9
	12.5	1.77	39.6	33.7	3.15	42.7	91.4

**Table 2:** Electrochemical Tafel polarization parameters in the presence of various concentrations of ASMS.

Solution	C_ASMS(*v*/*v* %)_	E_corr_(mV)	βa (mV dec^−1^)	βc (mV dec^−1^)	i_corr_ (mA cm^−2^)	σ
**2 M H_2_SO_4_**	0.00	−469	139	144	7.11	0.00
	1.00	−460	104	148	2.66	62.5
	3.50	−444	95.9	147	2.05	71.1
	5.00	−458	89.6	131	0.98	86.2
	10.0	−457	88.8	136	1.47	79.4
	12.5	−454	96.5	168	2.17	69.5
**2 M H_3_PO_4_**	0.00	−513	149	157	4.11	0.00
	1.00	−504	115	150	1.58	61.6
	3.50	−476	104	144	0.96	76.6
	5.00	−469	83.1	136	0.43	89.6
	10.0	−473	107	175	0.72	82.6
	12.5	−466	86.2	155	0.67	83.8

**Table 3:** Adsorption and activation thermodynamic parameters in the presence of a 5% concentration of ASMS.

T °k	Parameter	2 M H_3_PO_4_		2 M H_2_SO_4_	
		Free	with 5% ASMS	Free	with 5% ASMS
	K_ads_ (L/mL)	1.66	1.66		3.33
	ΔG_ads_ (kJ/mol)		−11.2		−12.9
	ΔH_ads_ (kJ/mol)		−52.7		−100
	Δ S_ads_ (J/mol)		−139		−293
	Ea (kJ/mol)	24.2	80.6	42.7	79.7
	ΔH* (kJ/mol)	21.9	77.9	40.1	77.1
	ΔS* (J/mol)	−160	11.3	−92.8	14.4
298	ΔG* (kJ/mol)	69.7	74.6	67.7	72.9
308		71.3	74.5	68.6	72.7
318		72.9	74.4	69.5	72.6
328		74.6	74.3	70.5	72.4

**Table 4:** The chemical composition of ASMS.

Compound Name	Compound Structure
Stigmasterol	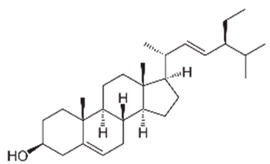
Cholesterol	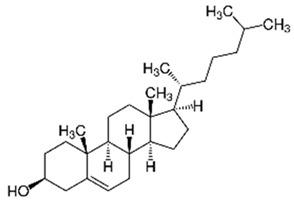
β-sitosterol	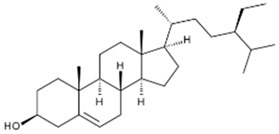
Heneicosane	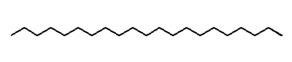
Timnodonic	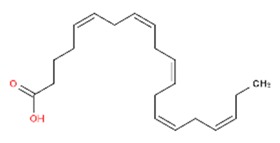
Linoleic acid	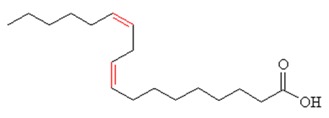
Oleic acid	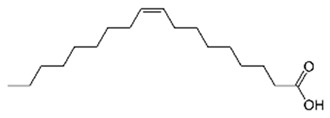

## Supplementary Materials

The following are available online at https://www.mdpi.com/1996-1944/12/18/3013/s1, Figure S1: Variation of surface coverage with temperature plots for carbon steel in two acids in presence 5% ASMS. Figure S2. Transition state plots for carbon steel in two acids in absence and presence 5% ASMS.
