# Supplementary material for: Comparative Evaluation of Anticorrosive Properties of Mahaleb Seed Extract on Carbon Steel in Two Acidic Solutions
Source: Materials (Basel). 2019 Sep 17;12(18):3013. doi: 10.3390/ma12183013 (PMC6766338; doi:10.3390/ma12183013)
Supplement: Supplementary file 1 [file materials-12-03013-s001.pdf]

Article

Comparative Evaluation of Anticorrosive Properties of Mahaleb Seed Extract on Carbon Steel in Two Acidic Solutions

Aisha A. Ganash

Chemistry Department, Faculty of Science, King Abdulaziz University, Jeddah Post code23714, Saudi Arabia; aganash@kau.edu.sa

Received: 10 August 2019; Accepted: 13 September 2019; Published: 17 September 2019

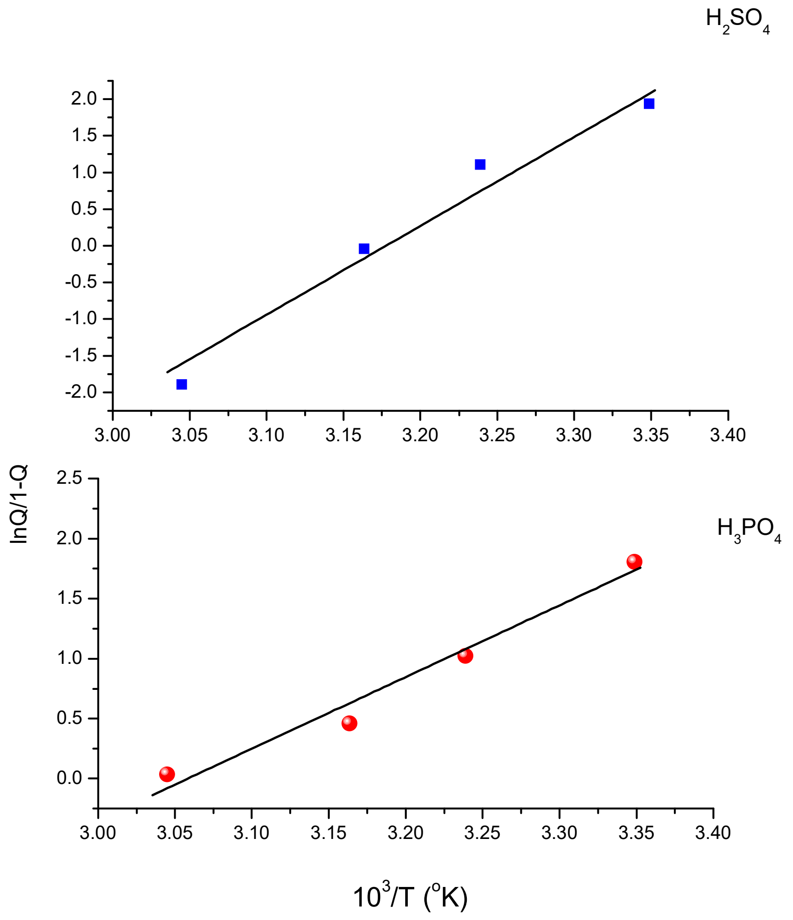

Figure S1. Variation of surface coverage with temperature plots for carbon steel in two acids in presence 5% ASMS.

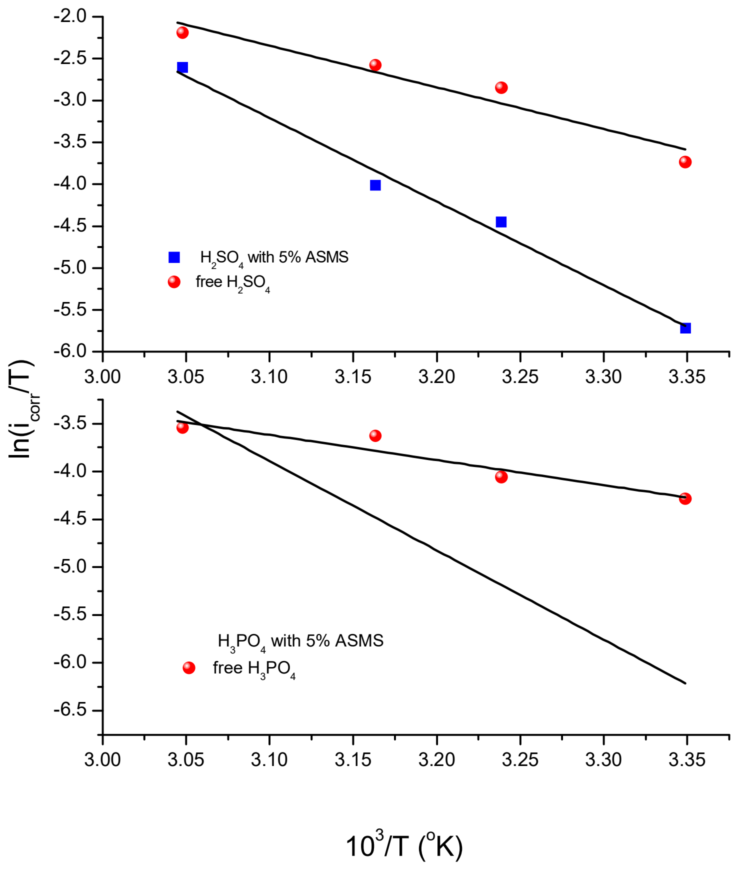

Figure S2.: Transition state plots for carbon steel in two acids in absence and presence 5% ASMS
